# The association between eating behaviour and moral views on animals from childhood to adulthood

**DOI:** 10.1111/bjdp.70057

**Published:** 2026-06-23

**Authors:** Tina Bagus, Noemi Thiede, Luke McGuire, Nadira S. Faber, Hanna Beißert

**Affiliations:** ^1^ Department of Teacher and Teaching Quality DIPF | Leibniz Institute for Research and Information in Education Frankfurt am Main Germany; ^2^ IDeA Center for Individual Development and Adaptive Learning Frankfurt am Main Germany; ^3^ Department of Comparative Cultural Psychology Max Planck Institute for Evolutionary Anthropology Leipzig Germany; ^4^ Department of Psychology University of Exeter Exeter UK; ^5^ Department of Psychology University of Bremen Bremen Germany; ^6^ Uehiro Oxford Institute University of Oxford Oxford UK; ^7^ Department of Elementary and Primary Education Goethe University Frankfurt Frankfurt am Main Germany

**Keywords:** cognitive conflict, human‐animal intergroup relations, meat‐eating, moral development, social norms, speciesism

## Abstract

How are moral judgements about animals related to whether people eat meat? We investigate the developmental trajectory of this question in German participants aged 8 to 74 years (*N* = 571, *M* = 24.84, SD = 14.52, female *n* = 394, male *n* = 177). Applying Bayesian modelling, we find no differences in moral judgements about animals and meat‐eating when comparing children who eat and who do not eat meat. With increasing age, however, we observe differences between dietary groups: while omnivorous participants show increasing speciesist beliefs and evaluate meat‐eating as morally acceptable, vegetarians' and vegans' moral judgements remain lower in speciesism and opposed to meat‐eating across age. Early adolescence is highlighted as a crucial phase in which eating behaviour and moral judgements start to align. In line with recent theoretical models, we discuss the role of diet in developing speciesism and its implications for the uptake of sustainable diets.

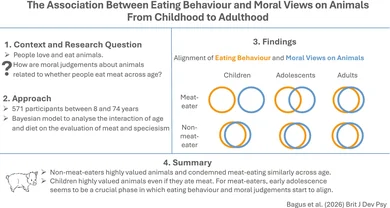

**Statement of Contribution**

**What is already known on this subject?**

- While adults' eating behaviour often aligns with their moral views on animals, less is known about this association in childhood and adolescence.
- Existing research indicates that children highly value animals even if they eat meat.
- A recent theoretical model proposed a developmental gap between eating behaviour and moral thinking about animals (Piazza et al., 2023).

**What does the present study add?**

- Our results provide the first empirical evidence for the proposed model. Children highly valued animals and condemned meat‐eating regardless of whether they ate meat or not.
- Vegetarians and vegans highly value animals and condemn meat‐eating similarly across all ages.
- For meat‐eaters, early adolescence seems to be a crucial phase in which eating behaviour and moral judgements start to align.

## INTRODUCTION

Humans have a complex psychological relationship with animals: at least in adulthood, some species are beloved, while others are eaten. One interesting puzzle is how the behaviour of eating animals is associated with moral judgements and, crucially, how this association varies over the course of development.

Most children are socialized into a world of human superiority over animals. These children grow up in societies where meat‐eating is the norm, part of long‐established food traditions and supported by parental and societal narratives about the necessity of eating meat (Joy, 2020; Roth, 2005). While younger children's diets are mostly determined by adults, this changes with age as adolescents have increasing autonomy (Neufeld et al., 2022; Story et al., 2002). Adults largely align their eating behaviours and their moral judgements about animals (Ioannidou et al., 2023; Piazza et al., 2015), whereas children simultaneously eat animals *while at the same time* judging meat‐eating to be wrong (Bagus et al., 2025; McGuire, Bagus, et al., 2025; Piazza et al., 2023). Therefore, childhood offers a unique opportunity to study the relation between behaviour and moral judgements. The present study investigates (a) developmental windows of greatest change in moral judgements about eating animals and (b) the role of diet in these judgements.

Recent research has documented that (Western) children value animals more than adults do (McGuire, Bagus, et al., 2025). For example, children aged 9–12 years think it is less morally acceptable to eat animals and reference animal welfare more frequently when reasoning about their evaluation (Bagus et al., 2025; McGuire, Fry, et al., 2023). Similarly, in moral dilemmas, 5‐ to 9‐year‐old children in the US and Poland show less pro‐human bias than adults (Paruzel‐Czachura et al., 2024; Wilks et al., 2021). These findings align with research on the development of *speciesism* (Caviola et al., 2019; Singer, 1975, 2023), where children are less likely to endorse the idea of a species‐based moral hierarchy (Caviola et al., 2025; McGuire, Palmer, & Faber, 2023). Initial evidence from the UK points to adolescence as a period where speciesism may even be higher than in adulthood (McGuire, Fry, & Faber, 2025). So far, however, the window of greatest change in speciesism remains unknown as no study to date has examined speciesism and related judgements about animals across the lifespan in a sample including children, adolescents and adults.

To better understand these developmental trajectories of judgements about animals, we must also consider the association between moral judgements and *eating behaviour*. Through socialization processes in dominant meat‐eating cultures, people learn that eating meat is ‘natural’, ‘necessary’ and ‘normal’ (Joy, 2020). These social narratives comprise beliefs about humanity's ancestors eating mostly meat, the physiological need for animal protein and widespread social expectations to eat meat (Joy, 2020). Piazza et al. (2015) added ‘nice’ as a fourth category of arguments for justifying meat‐eating and showed that a stronger endorsement of these justifications is related to lower moral concern for animals (Piazza et al., 2015). This rationalization of meat consumption serves as one way to navigate the conflict that emerges when people think about their condemnation of animal suffering while at the same time eating meat (Loughnan et al., 2014). Such conflicts can be reduced by *aligning* one's judgements and behaviours, as outlined in Dissonance Theory (Festinger, 1957; Rothgerber, 2020). Adults are particularly adept at adjusting their judgements to align with their behaviour, suggesting that to some degree behaviour may shape moral judgements (Bastian & Loughnan, 2017; Ioannidou et al., 2023; Rothgerber, 2020). For example, eating meat leads to reduced moral concern and denial of animals' minds (Bastian et al., 2012; Loughnan et al., 2010).

Conversely, individuals can reduce psychological conflict by adjusting their behaviour to align with their judgements (Festinger, 1957). Ethical vegetarians or vegans (from hereon veg*ns) are one example of a group who may have adjusted their behaviour to align with their moral judgements deviating from the social norm of meat‐eating. Veg*ns primarily cite moral, value‐led motives for taking up their diet and refer to animal rights or environmental concerns (Dhont & Ioannidou, 2024; Janssen et al., 2016; North et al., 2021; Rosenfeld, 2018). In adulthood, behaviours and attitudes can be mutually reinforcing so that cognitive validation of one's behaviour and behavioural validation of one's moral values reinforce dietary choice or views on animals' moral worth (Joy, 2020).

However, less is known about the relation between behaviour (diet) and moral judgements about animals in childhood and adolescence. This is crucial since recent work has documented that children who eat meat may still evaluate meat‐eating as morally wrong (Bagus et al., 2025; McGuire, Fry, et al., 2023). Piazza et al. (2023) argue that in societies where meat‐eating is customary at home, the disconnection between children's eating behaviour and their moral values about animals may be explained by limited knowledge about food systems. Without this knowledge or personal control of their diet, children may be less likely to experience conflict, and therefore their behaviour and judgements may not need to align. In adolescence, it is proposed that eating behaviour, food systems knowledge and moral values become increasingly connected, leading to an emerging meat‐related conflict (Piazza et al., 2023). At the same time, adolescents gain some control over their diet, e.g., when eating with their friends or making autonomous food choices outside the family context (Neufeld et al., 2022; Story et al., 2002). It is during adolescence that reasoning strategies begin to reference ideas that rely on knowledge about food systems—for example, arguments about ethical slaughter practices (Bagus et al., 2025; McGuire, Bagus, et al., 2025), stressing that this developmental window may be crucial for the association between behaviour and judgement.

While many adolescents and adults adjust their beliefs to align with their omnivorous diet, others do change their diet, including some children (Piazza et al., 2023). While limited, research examining vegetarian children's motives for abstaining from meat has shown that moral judgements and behaviour can also align in childhood. For example, vegetarian children from omnivorous families provide more moral reasons for not eating meat compared to omnivorous children but also compared to vegetarian children from vegetarian families (Hussar & Harris, 2010). So, existing studies with independent veg*n children and omnivorous children have shown similarities and differences in their reasoning about meat. As yet, there is no single study where diet, age and judgements are all included to examine the association of behaviour and judgements across development.

Taken together, in adulthood there is often an alignment of eating behaviour and moral judgements about animals, while this association is less consistent in childhood. Therefore, investigating this issue across a wide age range from childhood to adulthood offers a unique opportunity to study when such behaviour and moral judgements may begin to more closely align. Drawing on data from 8‐ to 74‐year‐old German participants, our first aim is to determine developmental windows of greatest change in moral judgements. Based on previous studies (McGuire, Fry, et al., 2023; McGuire, Palmer, & Faber, 2023), we assume that, with age, people will judge eating animals as more acceptable and speciesism will increase. Our second aim is to understand the association between behaviour (in this case, diet) and moral judgements. Specifically, we aim to understand at what ages judgements about animals diverge or align for veg*n and omnivorous participants.

## METHOD

### Open science

The study was approved by the ethics board of the DIPF | Leibniz Institute for Research and Information in Education in Frankfurt, as well as by the Ministry of Rhineland‐Palatinate. We report on the statistical sufficiency of our sample size, describe all data exclusions, provide detailed descriptions of all measures in the study and follow the Journal Article Reporting Standards (Appelbaum et al., 2018). Data and study materials have been made publicly available at the Open Science Framework and can be accessed at (https://osf.io/gmuew/overview?view_only=9b54263dd01b4c0d875b66f031a03f7c).

### Participants

A total of 665 German participants responded to the survey. Forty‐two participants were excluded because they did not complete the survey. As part of an initial check of the data, one further participant was excluded who reported that they were 99‐years‐old and still in work. Given this unlikely combination, we removed this participant. A missing data analysis indicated that the amount of missing data was 0.46%. Using the R package ‘naniar’, an additional Little's MCAR test (1988) indicated that data was missing completely at random, *χ* ^2^(41) = 53.7, *p* = .088. For complete case analysis, only participants that did not have any missing values in the relevant variables were considered for statistical analysis. Additionally, explanatory variables needed to be in binary format, e.g., *gender* [female; male]. Consequently, participants with any missing values or values that did not fit into binary format (e.g., any other gender identity) had to be excluded from the dataset.

This left a final sample of 571 participants aged from 8 to 74 years (*M* = 24.84, SD = 14.52, female *n* = 394, male *n* = 177). A post‐hoc visual inspection of the overlap of the prior and posterior distributions for the main effects of *age*, *diet* and their interaction revealed that the final sample size was sufficiently large for the model to provide well‐informed estimates (see Figures S1 and S2).

Most participants identified their ethnic identity as only German (76.4%) and 23.6% as having an ethnic identity other than or in addition to German. 60.6% of participants were members of a religious group, whereas 39.5% stated they were not religious. Most of the sample followed an omnivorous diet (74.3%, *M*_age_ = 24.21, SD = 15.44), while 25.7% followed a veg*n diet (*M*_age_ = 26.64, SD = 11.33). See Table S1 for detailed sample description for omnivore and veg*n participants. Half of the participants reported that they lived with a pet (50.3%).

### Procedure and materials

Adult participants were recruited through social media (e.g., Facebook, Instagram) and completed the online survey on via the survey software REDCap in 2022. To address our research questions, we also specifically distributed the questionnaire in forums for vegetarian and vegan people (e.g., vegetarierforum.de) in order to recruit a more balanced dietary sample. Participants volunteered and were not compensated for their time.

Children's and adolescents' data was collected in 2024 in schools in the Southwest of Germany via paper‐pencil format. Data collection with children and adolescents took place in a classroom setting. In primary school (8‐ to 10‐year‐olds), a team of two trained research assistants led the survey session and read out the instructions and questions of the first part of the survey. Children wrote their responses to the questions on their own questionnaires. For the second part, children completed the questionnaire independently, while the research assistants were on hand to answer questions and offer support. In secondary school (11‐ to 18‐year‐olds), participants were given instructions by a trained research assistant and filled out the questionnaire on their own.

All participants provided informed consent. Parental consent and participants' assent were obtained for participants below 16 years. Participants were presented with demographic questions about age, gender, ethnic identity, pet guardianship and diet. Following the assessment of demographic data, participants were asked to indicate how okay or not okay it is to eat animals (1 = *really not okay*; 6 = *really okay*; McGuire, Palmer, & Faber, 2023). This was followed by a German translation of a speciesism scale (Caviola et al., 2019; McGuire, Palmer, & Faber, 2023). The scale included six items, answered on a seven‐point Likert scale from 1 = *strongly disagree* to 7 = *strongly agree*. A child version of the speciesism scale (α = .78) was used for children's and adolescents' data collection in schools while the adults' version of the speciesism scale (α = .81) was used for the adults' online survey. The content of the scales was the same, but the wording of the items differed slightly, e.g., ‘Animals are worth less than humans’ in the child version (McGuire, Palmer, & Faber, 2023) and ‘Morally, animals always count for less than humans’ in the adult version of the scale (Caviola et al., 2019). All versions of the speciesism scale as well as a full version of the questionnaire with additional measures (unmotivated harm task, animal treatment task, categorization task) can be found on OSF.

### Statistical analysis

All analyses were conducted in R version 4.5.0 (R Core Team, 2025). Models were fitted via Bayesian inference using Hamiltonian MCMC, as implemented by the Stan inference engine (Stan Development Team, 2022), using the R package ‘brms’ (Bürkner, 2017). The scripts to conduct the analyses are accessible on OSF.

We specified two models, the first to analyse participants' meat evaluation and the second to examine speciesism. To formulate the models, we chose regularizing priors that are inherently conservative to ensure sufficient model stability. Specifically, we used exponential priors (SD = 10) for the standard deviation of the Gaussian Process (GP) terms (i.e., *age* and its interaction with *diet*) and inverse gamma priors (1.6083562, 5*0.073606) for the length scales (i.e., ‘wiggliness’) of the GP terms. For the fixed effects we used normal priors. Based on inspections of the impact of different priors on the outcome, we included *N*(0, 0.75) for most main effects and *N*(0, 0.375) for main effects where there was a small chance for complete separation.

To identify the model with the highest predictive accuracy, we compared the following models, respectively: GP model (i.e., *age* implemented as GP), GP model with interaction (i.e., two‐way interaction between *diet* and *age*), model with *age* as linear effect (with and without the *age‐diet* interaction), model excluding *age* and a GP model only including *age* (i.e., excluding *diet*, *gender, religion, pets*). For both the meat evaluation and speciesism model, loo‐comparisons indicated that the GP model including the interaction between *age* and *diet* was the best fit to the data.

To analyse the impact of *age* [8–74], *diet* [omnivore; veg*n] and their two‐way interaction on participants' *meat evaluation* score and on their rounded mean *speciesism* score, we, respectively, fitted an ordinal response model using a normal cumulative distribution (Bürkner & Vuorre, 2019). In both cases, we included *gender* [female; male], *religion* [non‐religious; religious] and *pet guardianship* [no; yes] as additional explanatory variables.

Before fitting both models, we scaled the continuous predictor *age* to a mean of 0 and a standard deviation of 1. All effects of *age* were implemented as Gaussian Processes, allowing the threshold parameters as well as the effect of *diet* to vary continuously and smoothly with *age* without assuming either a linear or fixed non‐linear shape to any of this variation. The sample for both models included 571 individuals. Both models converged (Rhat ≤1.01); their MCMC chains had sufficiently high resolution and they fit the data well as judged by posterior predictive plots (see Figures S3 and S4).

To investigate at what age we observe the steepest increase in *meat evaluation* and *speciesism* between 8 and 25 years of age we simulated a data set in which we held all variables but *age* and *diet* constant at 0 to average out their effects (i.e., *gender*, *religion*, *pets*). Subsequently, we inspected the predictions of the models per diet group regarding the probability to rate meat‐eating as morally okay versus not okay (i.e., the relative probability to rate 4–6 vs. 1–3) and the probability to be *more* rather than *less* speciesist (i.e., the relative probability to have a speciesism score of 3–7 vs. 1–2). In both cases, we identified the age range in which this probability increased most rapidly.

## RESULTS

### Interaction of age and diet on meat evaluation and speciesism across the lifespan

The effects of *age* on *meat evaluation* and *speciesism* varied depending on participants' *diet*. From an age of 10.18, omnivorous participants were more likely to rate meat‐eating as morally okay (vs. not okay; see Figure 1). In contrast, for veg*n participants, the estimated meat evaluation ratio remained mostly below 1 across ages (i.e., maximum ratio is 0.22 [0.08, 1.03]), indicating that participants were more likely to condemn meat‐eating regardless of age (see Figure S5 for meat evaluation ratio and speciesism ratio across the lifespan).

**FIGURE 1:**
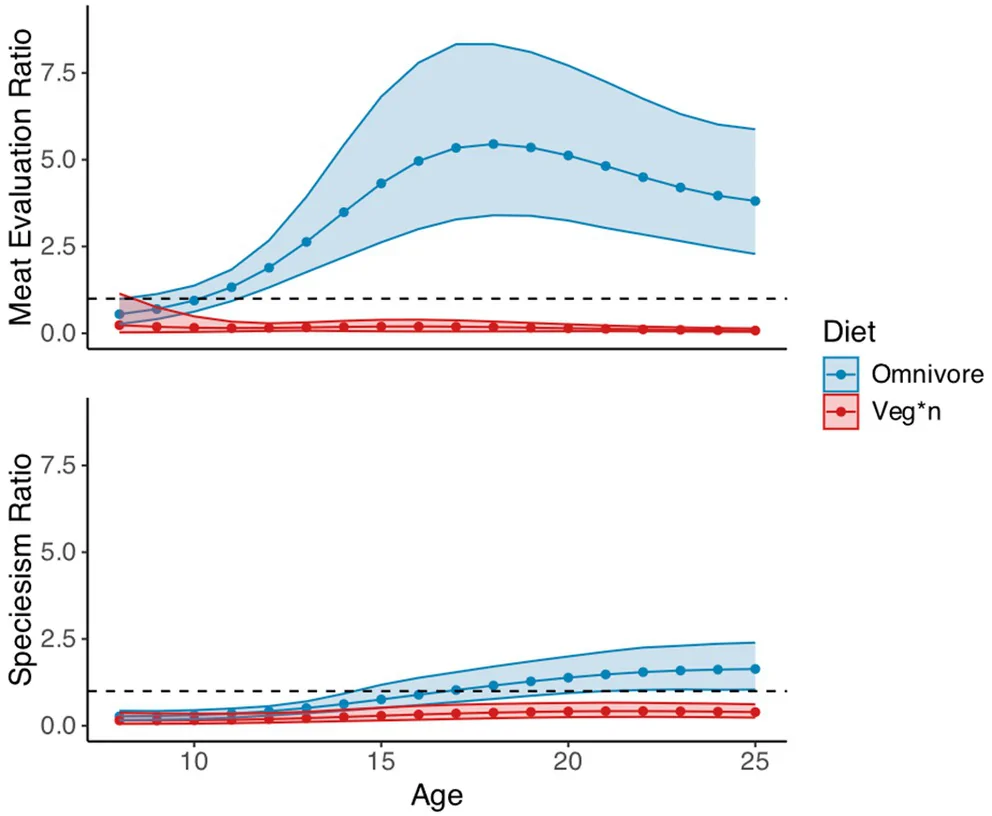
Meat evaluation ratio and speciesism ratio for 8‐ to 25‐year‐olds separated by diet. The lines represent the posterior mean for each age per *diet*, marginalizing over *gender*, *pets* and *religion*, while the shaded areas indicate the 95% credible intervals. For the meat evaluation ratio, the horizontal line at *y* = 1 indicates equal probability of evaluating meat‐eating as morally okay (4–6) versus not okay (1–3). For the speciesism ratio, the horizontal line at (*y* = 1) indicates equal probability of being more (3–7) vs. less (1–2) speciesist.

Regarding *speciesism*, from an age of 16.85, omnivores were more likely to report higher rather than lower speciesism (i.e., have a speciesism score of 1 or 2 as opposed to 3–7). In contrast, participants following a veg*n diet were more likely to report lower speciesism scores across ages (i.e., maximum ratio is 0.51 [0.25, 1.54]). Interestingly, the difference between dietary groups was larger regarding *meat evaluation* compared to *speciesism*.

### Developmental window of greatest change in meat evaluation

Inspecting the change in the ratio of evaluating meat‐eating as morally rather okay than not okay (i.e., the relative probability to give a rating of 4–6 vs. 1–3) across the age range of 8 to 25 indicated that the steepest increase in agreement to meat‐eating occurred around age 14 (*M* = 13.85, 95% CI = [9.5, 24.03]). The extent of that increase varied substantially depending on participants' *diet* (see Figure 1). Whereas for omnivorous participants the estimated increase in meat evaluation ratio was 3.27 ([2.05, 4.92]) within the estimated developmental window of steepest change, for veg*n participants this ratio did not change much and even slightly decreased −0.08 ([0.02, −0.51]).

### Developmental window of greatest change in speciesism

Inspecting the change in ratio of being more rather than less speciesist (i.e., the relative probability to have a speciesism score of 3–7 vs. 1–2) in the age range of 8 to 25 revealed a general increase across age. The steepest point of increase was observed around age 17 (*M* = 16.83, 95% CI = [13.5, 22.5]). Like the meat evaluation model, changes in speciesism across age varied depending on participants' *diet* (see Figure 1). Within that estimated developmental window of greatest change, the estimated increase in speciesism ratio for omnivorous participants was 1.06 ([0.71, 1.54]) and for veg*n participants 0.20 ([0.14, 0.24]).

### Further demographics

We observed very strong evidence for the effects of *gender* and *religion on meat evaluation*: being male and being religious were positively associated with rating meat‐eating as morally okay. Moreover, there was moderate evidence that living with pets reduced the probability of rating meat‐eating as morally okay (for details, see Table S2).

Further, we observed very strong evidence for the effects of *gender*, *religion* and *pet guardianship* on *speciesism*. Living with pets was negatively associated with *speciesism*. In contrast, being male and being religious were positively associated with being more speciesist (for details, see Table S3).

## DISCUSSION

The present study examined the interplay of eating behaviour and moral judgements about animals across the lifespan, aiming to determine developmental windows of greatest change in these moral judgements. As hypothesized, age effects varied as a function of participants' diet: While meat evaluation and speciesism levels were similarly low amongst different dietary groups in childhood, the judgements of omnivores and veg*ns began to diverge from early adolescence.

### The role of diet in the development of moral judgements about animals

Existing research has suggested that with age, speciesism increases and people become more likely to deem eating animals as acceptable (McGuire, Palmer, & Faber, 2023). The present study aimed to extend this research by considering participants' diet. Consistent with previous findings (McGuire, Bagus, et al., 2025; McGuire, Palmer, & Faber, 2023), our results indicated that children showed lower speciesism and were less likely to think that eating animals was morally acceptable, regardless of their diet. From there, speciesism and positive evaluations of meat‐eating increased with age for omnivorous participants. Beginning from an age of 10 to 11‐years, omnivorous participants evaluated eating animals as more morally okay and from 16 to 17‐years, they were more likely to report higher speciesism scores. Across age veg*n participants reported that it was not morally okay to eat meat and their speciesism remained low, with the differences between omnivores and veg*ns becoming more pronounced amongst older individuals. These findings may indicate that with age, eating behaviour plays an increasingly important role for moral judgements about animals.

In line with previous research on adults, our results point towards the resolution of meat‐related conflict through aligning of cognitions about animals' moral worth with eating behaviour and vice versa (Piazza et al., 2015; Rothgerber, 2020). That is, adults and adolescents seek consistency between their moral judgements and their (socialized) eating behaviour, which has been acquired in a society where meat‐eating is normative (Rothgerber, 2020). In childhood, meat‐eating is seen as less morally acceptable and speciesism tends to be lower. From early adolescence onwards, youth adjust their judgements. We take this to be initial evidence for the role of eating behaviour in guiding moral judgements about animals, which likely is—alone and in interplay with other socialization factors (Carter et al., 2026)— part of the mutually reinforcing nature of behaviour and judgements.

Our results provide empirical evidence for the proposed developmental gap between eating behaviour and moral thinking about animals (Piazza et al., 2023) as in middle childhood, diet seemed to play a minor role in children's moral judgements about animals. In contrast, adolescents' moral views on animals were more closely *aligned with* their meat‐eating behaviour. This is consistent with Piazza et al. (2023) which argues that adolescents' food systems knowledge becomes more accurate, leading to an emerging meat‐related conflict that needs to be resolved by either adjusting moral values (omnivorous participants) or eating behaviour (veg*n participants). Endorsing beliefs about animals' lower moral status is one socially established route of reducing this conflict that serves as a means to rationalize meat‐eating (Bastian & Loughnan, 2017; Piazza et al., 2015).

Lastly, our data suggests that veg*n participants did not differ in their moral judgements about animals across the lifespan. That is, veg*n children and adults were equally likely to be low in speciesism and their negative evaluation of meat‐eating. One explanation consistent with existing theoretical models is that these veg*n participants both as children and as adults are less likely to experience meat‐related conflict. For veg*n children, this lack of conflict may be more like that of the omnivorous children, in part due to a lack of knowledge, with their diet perhaps chosen for them by their parents. For veg*n adults, this may more likely follow a classic dissonance account, as eating behaviour (i.e., eating a vegetarian or vegan diet) is already aligned with individuals' beliefs about animals' moral worth (Piazza et al., 2023). These two processes may in part explain the lack of age‐related effects amongst veg*n participants, though future work here is required to uncover more about the conflict experienced by veg*n children, adolescents and adults. While our results can be interpreted against the backdrop of Piazza and colleagues' model of meat‐related conflict and the role of food systems knowledge, we acknowledge that there are alternative possible accounts for our age‐related findings that ought to be probed in future research. While knowledge of food origins plays a key role in adults' moral judgements (Kunst & Hohle, 2016), other work demonstrates that some adults show gaps in their knowledge of factory farming practices (Feltz et al., 2022). While an understanding of the meat‐animal link and process knowledge may be developing, adolescents also undergo important developmental transitions. For example, peers exert a powerful influence on adolescents' moral judgements and behaviours (Busching & Krahé, 2020), even in the realm of food consumption (Ragelienė & Grønhøj, 2020; Stok et al., 2016). It may therefore be the case that the increasing speciesism and pro‐meat evaluations of adolescents seen here reflect transmission of such attitudes within peer groups. As part of spending more time outside the family home, dietary autonomy likely increases, leading to an increased need to take responsibility for one's food choices and justify these within one's peer group, which may in turn lead to the uptick in speciesism seen in this and other work with adolescents. Such an account is consistent with findings from studies on reasoning about eating animals that point towards adolescence as a transitional phase during which adolescents begin referring to ‘adult‐like’ justifications like social conventions about meat‐eating (Bagus et al., 2025; McGuire, Fry, & Faber, 2025). Both this plausible account and that of Piazza et al. (2023) are consistent with a dissonance or conflict reduction argument and as such further work is needed to understand both the trigger points and the degree of adolescents' felt conflict around meat and animal treatment.

### Developmental windows of greatest change

The investigation of developmental windows of greatest change revealed the greatest increase in meat evaluation around age 14 and the greatest increase in speciesism around age 17, adding further evidence for adolescence as a crucial period of change in moral judgements about animals (McGuire, Fry, & Faber, 2025). Here, we observe substantial changes in omnivorous adolescents, while there is little to no change in veg*n participants.

Interestingly, the developmental window of greatest change in meat evaluation is earlier in development than the estimated greatest increase in speciesism. While urging caution in overinterpreting these descriptive differences, one possible explanation is that youth learn first about meat‐eating as a concrete behaviour and then extend outwards to the more abstract concept of human domination over animals. Evaluating meat‐eating as more acceptable with age reflects a concrete justification of one's behaviour as compared to the more abstract notion of animals' moral worth. This, in turn, might explain why the difference between diet groups is even more pronounced for meat evaluation than for speciesism.

### Limitations and future directions

While our study focused on developmental trajectories, our results are based on a cross‐sectional design that does not allow us to draw conclusions about causality. Thus, longitudinal research that examines intrapersonal changes in moral judgements about animals over time, in the crucial period between late childhood and early adolescence, will be an important contribution to the field.

Our results were interpreted in line with the developmental model of meat‐related conflict (Piazza et al., 2023) that posits food systems knowledge as a potential factor driving the shift in moral views on animals during adolescence. We therefore encourage future research to investigate the development of food systems knowledge from middle childhood to late adolescence to understand its role in the occurrence of meat‐related conflict as a driver for diet‐specific changes in moral views on animals.

Additionally, we acknowledge that our sample is from only one nation that is considered a WEIRD (Western, Educated, Industr ialized, Rich, Democratic) population. Recent research has indicated that developmental trajectories in the moral view on animals vary across different cultural contexts (Hopwood et al., 2025; Neldner et al., 2026). Thus, further cross‐cultural research is needed to replicate our findings in more diverse contexts.

Efforts to mitigate climate change will in part rely on a global shift towards plant‐based diets (Godfray et al., 2018; Hahn et al., 2021), with attempts to promote such diets finding limited success in adults (Green et al., 2026). We hope our findings demonstrate that future research ought to consider these windows of developmental change from pre‐adolescence as a crucial period in which youth shape their moral minds about animals and interventions in the direction of more sustainable human‐animal relations may be fruitful.

## CONCLUSION

For adults, moral judgements about the worth of animals are powerfully intertwined with behaviours that involve the use of animals. Our results point towards adolescence as a crucial period for the development of moral judgements related to meat‐eating. We argue that omnivorous adolescents' increase in speciesism and tendency to find meat‐eating more acceptable relates to the emergence of meat‐related conflict that urges adolescents to bring their moral judgements about animals in alignment with their eating behaviour. Our findings suggest focusing on tailored target groups for value‐led educational programmes on human–animal interactions, particularly to support adolescents during their critical phase of forming moral judgements about animals.

## AUTHOR CONTRIBUTIONS

**Tina Bagus:** Conceptualization; methodology; data curation; investigation; formal analysis; writing – original draft; project administration; resources. **Noemi Thiede:** Conceptualization; methodology; writing – review and editing; visualization; formal analysis; investigation. **Luke McGuire:** Conceptualization; methodology; supervision; writing – review and editing. **Nadira S. Faber:** Conceptualization; methodology; writing – review and editing. **Hanna Beißert:** Conceptualization; methodology; supervision; resources; writing – review and editing.

## FUNDING INFORMATION

This research did not receive any specific grant from funding agencies in the public, commercial or not‐for‐profit sectors.

## CONFLICT OF INTEREST STATEMENT

The authors declare no conflicts of interest.

## ETHICS STATEMENT

The study received ethical approval from the ethics committee of DIPF | Leibniz Institute for Research and Information in Education.

## Supporting information


Table S1
Table S2
Table S3
Figure S1
Figure S2
Figure S3
Figure S4
Figure S5

## Data Availability

The data and code necessary to reproduce the analyses presented here are publicly accessible, as are the materials necessary to attempt to replicate the findings. Data, code and materials for this research are available at the following link: https://osf.io/gmuew/overview?view_only=9b54263dd01b4c0d875b66f031a03f7c. The analyses presented here were not preregistered.
