# Supplementary material for: The association between eating behaviour and moral views on animals from childhood to adulthood
Source: Br J Dev Psychol. 2026 Jun 23;44(4):920–31. doi: 10.1111/bjdp.70057 (PMC13648304; doi:10.1111/bjdp.70057)
Supplement: Supplementary file 1 [file BJDP-44-920-s001.docx]

**Supplementary Material**

**Table S1**

*Sample description per diet*

|  | Gender  male \| female | | Religion  religious \| not religious | | Pet guardianship  pet \| no pet | | Age  *M (SD),* range |
| --- | --- | --- | --- | --- | --- | --- | --- |
| Omnivore | 150 | 274 | 291 | 133 | 207 | 217 | 24.21 (15.44), 8-74 |
| Veg*n | 27 | 120 | 55 | 92 | 80 | 67 | 26.64 (11.33), 8-59 |
| Total | 177 | 395 | 346 | 225 | 287 | 284 | 24.84 (14.52), 8-74 |

**Table S2**

*Results of the meat evaluation model*

| Effect | Posterior Mean | lower CI | upper CI | Directional Certainty |
| --- | --- | --- | --- | --- |
| Diet | -0.47 | -1.24 | 0.26 | 0.89 |
| Gender | 0.63 | 0.29 | 0.97 | 1.00 |
| Pets | -0.17 | -0.47 | 0.13 | 0.87 |
| Religion | 0.30 | -0.03 | 0.63 | 0.96 |

*Note.* As we modelled an interaction between *diet* and *age*, the effect for *diet* as displayed in the table is the expected effect for averaged aged participants (mean age = 24.84). CI = credible interval.

**Table S3**

*Results of the speciesism model*

| Effect | Posterior Mean | lower CI | upper CI | Directional Certainty |
| --- | --- | --- | --- | --- |
| Diet | -0.66 | -1.39 | 0.14 | 0.95 |
| Gender | 0.86 | 0.52 | 1.20 | 1.00 |
| Pets | -0.52 | -0.83 | -0.22 | 1.00 |
| Religion | 0.43 | 0.11 | 0.76 | 1.00 |

*Note.* As we modelled an interaction between *diet* and *age*, the effect for *diet as displayed in the table* is the expected effect for averaged aged participants (mean age = 24.84). CI = credible interval.

**Figure S1**

*Meat evaluation: Overlap of prior and posterior distribution for central model parameters*

**Figure S2**

*Speciesism: Overlap of prior and posterior distribution for central model parameters*

**Figure S3**

*Posterior Predictive Check - Meat Evaluation Model*

*Note.* The observed distribution of meat evaluation responses (black line) is compared to 70 replicated datasets drawn from the posterior predictive distribution (light grey lines). The observed data fall well within the range of model-predicted distributions, suggesting adequate absolute model fit.

**Figure S4**

*Posterior Predictive Check - Speciesism Model*

*Note.* The observed distribution of speciesism responses (black line) is compared to 70 replicated datasets drawn from the posterior predictive distribution (light grey lines). The observed data fall well within the range of model-predicted distributions, suggesting adequate absolute model fit.

**Figure S5**

*Meat evaluation ratio and speciesism ratio across the lifespan separated by diet*

*Note.* The lines represent the posterior mean for each age per *diet*, marginalizing over *gender*, *pets*, and *religion*, while the shaded areas indicate the 95% credible intervals. For the meat evaluation ratio, the horizontal line at y = 1 indicates equal probability of evaluating meat-eating as morally okay (4-6) vs. not okay (1-3). For the speciesism ratio, the horizontal line at (y = 1) indicates equal probability of being more (3-7) vs. less (1-2) speciesist.
